# Examining potential gender bias in automated-job alerts in the Spanish market

**DOI:** 10.1371/journal.pone.0260409

**Published:** 2021-12-10

**Authors:** Naroa Martínez, Aranzazu Vinas, Helena Matute

**Affiliations:** Departamento de Psicología, University of Deusto, Bilbao, Spain; Universitat Jaume I, SPAIN

## Abstract

Numerous field experiments based on the correspondence testing procedure have documented that gender bias influences personnel selection processes. Nowadays, algorithms and job platforms are used for personnel selection processes because of their supposed neutrality, efficiency, and costs savings. However, previous research has shown that algorithms can exhibit and even amplify gender bias. The present research aimed to explore a possible gender bias in automated-job alerts generated in InfoJobs, a popular job platform in Spain. Based on the correspondence testing procedure, we designed eight matched resumes in which we manipulated the gender of the candidate for two different professional sectors (female-dominated vs. male-dominated) and two different levels of age (24 vs. 38). We examined the 3,438 offers received. No significant differences were observed in the automated-job alerts received by female and male candidates as a function of occupation category, salary, and the number of long-term contracts included in the alerts. However, we found significant differences between the female-dominated and the male-dominated sectors in all the mentioned variables. Some limitations and implications of the study are discussed. The data and materials for this research are available at the Open Science Framework, https://osf.io/kptca/.

## Introduction

Research in recent years has documented gender discrimination in job access, hiring decisions, selection of leaders, as well as in salaries [1–4]. In particular, it has been shown that female candidates have a higher probability of being selected for female-dominated and low qualified positions [5–7], and that less than 5% of executive positions are held by women [8]. In addition, financial disparities between men and women are increasing worldwide [9], and the gender pay gap is still around 16% in Europe [10]. This disparity is still present nowadays even though, according to the Organization for Economic Cooperation and Development [11], women are more likely to graduate from college than men.

Many causes contribute to gender inequality, but gender bias plays an important role in the workplace [12]. Due to well-known cognitive biases, people can often reach erroneous conclusions [13] and develop stereotypes and prejudices [14]. For example, female applicants are expected to be less self-confident, less likely to be committed, and less likely to stay in the job than male applicants [15]. Indeed, the International Labour Organization [16] recognizes that gender bias is one of the leading causes of discrimination in the hiring and promotion of workers with the same qualifications and merits.

For more than 40 years, field experiments studying discrimination in personnel selection processes have generally been carried out using the so-called correspondence testing procedure [17–22]. The correspondence testing procedure consists of submitting matched pairs of female-male resumes in response to job offers, to compare whether women are discriminated against during this process. Studies based on the correspondence testing procedure have traditionally been conducted face-to-face, over the telephone, or through written correspondence [17]. However, current technological development has changed the recruitment process, and job platforms that make use of algorithms have become the main hiring channel for most companies. For example, the database of InfoJobs, which is one of the most used job platforms in Spain [23], contains eight million resumes [24]. More than 550,000 companies have used this job platform, and more than 11 million contracts have been closed [25]. For this reason, researchers have also started to carry out studies in human recruiters based on the correspondence testing procedure through job platforms (e.g. [5] in Spain, [26] in France, [27] in China). However, as far as we know, these studies have not tested whether the algorithms used for recruitment themselves show the discrimination biases that are already documented in humans. That is precisely the purpose of our research.

Next, we will review two different lines of research. First, studies examining gender biases in humans using the correspondence testing procedure through a job platform. Second, the literature on gender biases when algorithms are used for recruitment. We highlight the need to link these two lines of research in order to examine gender bias in algorithms using the correspondence testing procedure.

### Gender biases in humans through job platforms

An interesting field experiment that made use of the correspondence testing procedure through a job platform was published by Albert et al. [5]. In this experiment, the authors aimed to analyze gender and age discrimination shown by humans in the labor market of Madrid, using InfoJobs. Among other findings, the authors observed clear evidence of discrimination in callback rates on the basis of age as well as a preference toward women in female-dominated positions (e.g., assistant, receptionist, secretary) and low qualified positions. These results were in line with other studies on gender discrimination, which showed that women were significantly more present in low-qualification positions, which, in turn, are usually female-dominated positions [21, 26–28]. It has also been documented that men need to submit two or three times more applications than women to get positive feedback on offers from female-dominated sectors (see [29] for a meta-analysis).

All of the studies using correspondence testing procedures through job platforms that we are aware of (e.g. [5, 26, 27]) have been conducted in human recruiters with the purpose of finding whether human biases exist during the short-listing phase. This phase involves an initial screening from the pool of applicants to select which will be placed on the shortlist. However, nowadays, the use of algorithms during the recruitment process is increasing. For example, there is a growing trend to use artificial intelligence algorithms to scan resumes [30] and video-interviews [31]. Moreover, personnel selection processes also involve the use of algorithms during the earliest stages of the selection process, that is, even before the short-listing phase. For example, algorithms are commonly used in job platforms to send the automated job alerts to candidates in a personalized way. These personalized and automated job alerts are supposed to enable access to the most relevant job offers for each candidate (e.g [23]), and it has been shown that algorithmic recommendations do affect human judgments and decisions in important areas such as politics and dating [32]. Therefore, these personalized recommendations may become a critical and influential point for each candidates’ job access, and they could be biased.

An interesting line of research could consist of studying potential biases in those algorithms, using procedures similar to those used in field experiments to study gender biases in humans. In particular, the usual procedure of correspondence testing could be applied to audit algorithms involved in the personnel selection process.

### Gender biases using algorithms for recruitment

There is a tendency to believe that dealing with a machine rather than with a human is more objective and rational, and is free of biases [33–35]. However, human biases are present during the algorithm life cycle, and systematic errors of algorithms can arise [36, 37]. For example, companies use “historical” data to train the algorithm so that algorithms can “learn” the patterns. This poses the risk of reproducing and amplifying the biases already present in our society and in our databases. Indeed, many previous studies have found that machine learning algorithms tend to reproduce human biases [38–40].

With regard to gender bias, research with algorithms is still at its early stages, but some findings are clear. For instance, Bolukbasi [38] found gender stereotypes in algorithms trained on Google News. These algorithms tended to associate nouns such as “brilliant”, “architect”, and “great”, more often with the word “he”, whereas they frequently associated the words “mom”, “housewife”, and “princess” with the word “she”. Similarly, other studies have reported that algorithms trained on a corpus of text from the Internet associate female names such as Sarah more often with family-related words such as “parents” and “wedding”, while male names such as John had stronger associations with career-related words such as ‘professional’ and ‘salary’ [39].

Concerning the recruitment process, in an automated experiment conducted by Datta et al. [41] the authors showed that setting the gender to female when using Google Ads resulted in getting fewer instances of an ad related to high-paying jobs than setting it to male. Amazon also had to remove its algorithm from the selection process because it showed gender biases [42]. Evidence of gender bias has also been found in algorithms of online communities that affect labor markets [43] and in Task Rabbit and Fiverr’s search algorithms, two prominent independent online markets [44]. In addition, Day [45] also reported that LinkedIn reflected a gender bias, with an algorithm suggesting male names (e.g. Stephen Williams) when searching for female candidates (e.g., Stephanie Williams). In the case of InfoJobs, polemic discussions on potential gender bias in the automated-job alerts that they send to candidates have occurred in social media networks. One user reported finding that changing the gender on the job platform from female to male resulted in better-qualified and better-paid job offers. This case reached national newspapers in Spain [46], but to our knowledge, this has not been systematically researched.

In sum, most of the field experiments based on the correspondence testing procedure have focused on the influence of human gender biases during the hiring process. However, the use of algorithms and job platforms in human resources departments are increasing (e.g [30]). Typically, algorithms and job platforms are broadly used during the initial personalized automated-job alerts phase (e.g. [47]). This initial step includes potentially relevant job offers and is critical for the candidates’ job access.

### The present study

The purpose of the present research was, therefore, to explore whether there was a gender bias in InfoJobs automated-job alerts. To this end, we used the correspondence testing procedure. In particular, we examined different characteristics of the offers received through the automated-job alerts (such as the occupation category, salary, and long-term contracts) as a function of whether gender was set as female or male in the resume of the candidate. Several factors that potentially could influence gender bias during the personnel selection process were controlled for: professional sector (female-dominated vs. male-dominated), and the candidate’s age.

## Materials & methods

**Fig 1:**
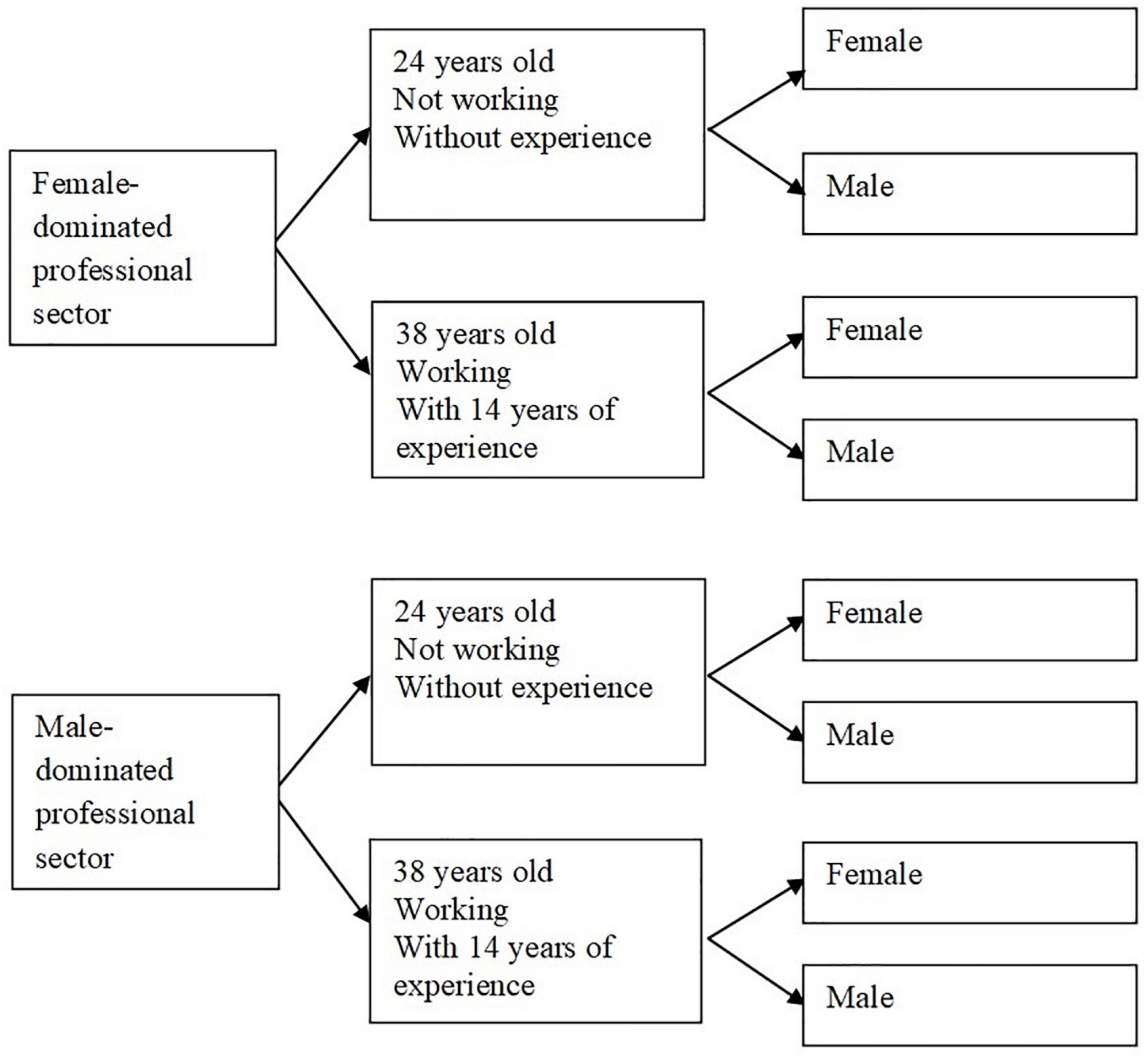
Design summary of the experiment: Eight resumes were matched-paired and submitted to the InfoJobs platform.

We used the InfoJobs platform to design and register eight matched resumes in which the gender of the candidate was manipulated (four resumes from women and four from men were matched-paired). In addition, the design of the experiment controlled for the professional sector (female-dominated vs. male-dominated) and age (24 vs. 38), as potentially confounding variables. We decided to reduce the number of matched resumes and the conditions to be analyzed to eight, in order to have a feasible design. Fig 1 summarizes the design of the experiment.

Table 1 summarizes the data entered during the registration process for each of the eight matched profiles. We entered gender as female for half of the candidates and male for the other half. To enter the first names and surnames of the candidates, we used the most popular names and surnames in Spain for both females and males, based on data from the Spanish National Statistic Institute, In Spanish “Instituto Nacional de Estadística”, INE [48]. We used María, Carmen, Ana, and Isabel for female profiles; David, Juan, Javier and, Daniel for male profiles. García, Gonzalez, Rodríguez, Fernández, López, Martínez, Sánchez, and Pérez were used as their surnames. Names and surnames were used at random. Our selection of the different Spanish names and surnames was intended to create ecological resumes while matching the features of names such as frequency and popularity. All names and surnames selected were Spanish, popular, and frequent in order to avoid introducing biases related to race, ethnicity, or any other minority groups that could be suggested by less frequent names [18]. Thus, we controlled for the possible effects of the candidates’ names in the hiring process since previous literature had pointed out that different names can lead to different hiring rates (e.g. [18]) and can affect the performance of artificial intelligence algorithms [39, 40].

**Table 1:** Data that we entered during the registration process of the eight matched profiles.

Requested data	Data
Personal data	
Name	Ana, Carmen, Isabel, María, Daniel, David, Javier, Juan
Surname	Fernández, García, González, López, Martínez, Pérez, Rodríguez, Sánchez
Date of birth	Age 24: 29/01/1995
	Age 38: 29/01/ 1981
Gender	Female
	Male
Do you live in Spain?	Yes
Postal code	28012
Professional experience	
Are you working?	Age 24: No
	Age 38: Yes
In case you are working, starting date	Age 24: Non-applicable
	Age 38: June 2005
Academic degrees	
Degree	Other
Name of degree	Other degrees, certifications and licences
End date	Age 24: June 2019
	Age 38: June 2005

When defining the age of the candidates, we used 24 and 38 years old, following the study by Albert et al. [5], which used 24, 28, and 38 years old. In our study, we decided to keep only the two extreme values of the three used in Albert et al. [5]. Our purpose was to examine the possible effect of gender while controlling for age and minimizing the number of conditions to be examined. Because information about employment status was mandatory, we combined the information regarding the candidates’ age with information about their current professional experience. We described the 24-year-olds as applicants without any experience and not working at the moment, and the 38-year-olds as active workers with 14 years of experience.

We then selected the professional sector on the InfoJobs options: “health” was selected as the female-dominated professional sector and “engineers and technicians” as the male-dominated professional sector. These professional sectors were selected because they were the sectors with more women and men, respectively, according to the data of active population by gender and activity in 2017 in Spain [49] as well as from the Spanish Ministry of Science, Innovation and Universities [50]. According to these data, engineering and architecture are the degrees with more enrolled men, and health sciences include the ones with more enrolled women. Finally, the geographical area selected was Madrid because this is one of the areas receiving more job offers within Spain.

We also configured the minimum required fields to receive personalized and automated-job alerts. These are the professional sector of interest (“health” or “engineers and technicians”) and the geographical area (Madrid). According to InfoJobs, the automated-job alerts allow candidates to receive the job offers that best fit their profile [47].

### Ethical considerations

This article does not contain any studies with human participants or animals performed by any of the authors. In the context of the present research, any potential ethical problems related to the correspondence testing procedure [21] do not apply because we are not examining the biases of human participants but the biases of algorithms. Even so, we took some measures to minimize the possible impact that this could generate. Firstly, in no case did we register the fictitious candidates to any offer or send the fake resumes to any company or employer. Secondly, the profiles were not made visible, so they did not appear in search engines and could not be found by companies. In addition, only the minimum information required to receive job alerts was configured and in no case did we include documents or photos. Finally, the profiles were deleted after the study and we removed the company name information from the data file.

### Procedure

We collected the data of the automated-job alerts for the eight matched resumes during 28 days from the day of registration, March 5^th^, to April 1st, 2019. InfoJobs included a maximum of 18 offers in each daily automated-job alert. In order to compare the offers received, we examined occupation categories, salary information, and long-term contracts included in the automated-job alerts received by the matched-pairs of female-male resumes. We classified the offers received according to the occupation category as defined in the National Classification of Occupations (Clasificación Nacional de Ocupaciones, CNO-11) published by INE [51]. The occupation categories used by the INE are the same ones as those used by the International Labour Organization [52] and the European Commission [53]. CNO-11 [51] contains a total of 10 different occupation categories. We selected as target the first three occupation categories described by INE [51]: (1) managers, (2) professionals, and (3) technicians. This selection is based on the following criterion: Among the 10 categories of the CNO-11, only the first three occupation categories are represented in both of the selected professional sectors (“health” and “engineers and technicians”). For example, engineering positions are represented only at these first three occupation categories, while health positions cover up to the fifth occupation category. For this reason, only the first three occupation categories were selected as dependent variables. This allowed us to observe the possible effect of gender on the occupation category with a feasible experimental design. Based on the CNO-11 classification, two researchers independently categorized each offer according to its description as (1) managers, (2) professionals, (3) technicians, and (0) others and their independent classifications were compared. Offers classified as “others” corresponded to other occupation categories different from managers, professionals and technicians and offers including confusing descriptions which could not be properly classified. The percentage of agreement between judges was 91% (κ = 0.88), which is an excellent index of agreement [54].

### Variables

The variables computed in this study are described below.

#### Occupation category

As previously mentioned, for our analysis we used three different types of occupation categories as targets: (1) managers: offers that correspond (or not) to the CNO-11 occupation category of managers; (2) professionals: offers that correspond (or not) to the CNO-11 occupation category of professionals; (3) technicians: offers that correspond (or not) to the CNO-11 occupation category of technicians. In addition, offers that did not correspond to any of the three occupational categories mentioned above as well as offers including unclear descriptions were classified as missing values and were therefore not analyzed. Finally, offers in which there was no agreement between judges, were classified as missing values and were therefore not analyzed.

#### Annual gross salary

The annual gross salary corresponds to the number of euros calculated from the information provided in the offer as “annual salary”. In the majority of the offers (93% of offers for which salary was informed) the minimum and maximum salaries were specified and the average salary was calculated. In cases in which the reported amount was defined per month or per hour, the salary was estimated by multiplying it by 12 (corresponding to 12 months) or by 1,826 (corresponding to the number of working hours in the 2019 Spanish working calendar), respectively. In cases in which the reported amount was associated to part-time contracts we calculated the amount for a full-time salary. Those offers that did not report a salary were classified as missing values and were therefore discarded.

#### Number of long-term contracts

The long-term contract variable corresponds to whether the offer was marked as “indefinido” [long-term] or not in the description of the offer. Those offers that did not report temporariness information (duration of the contract) were classified as missing values and were therefore discarded.

## Results

We analyzed the automated-job alerts received in 24 days. We excluded four days from the analysis because they corresponded to Sundays, and on those days, very few offers were received (none in the female-dominated professional sector and a maximum of three offers in the male-dominated professional sector).

A total of 3,438 offers were received, corresponding to an average of 18 daily automated-job alerts in 24 days for the eight matched resumes (*M* = 430 offers per resume; *SD* = 6.36).

We examined the number of job offers received in each of the three occupation categories defined by INE [51] (see Table 1). Table 2 shows the total number of offers received by the candidates and classified by the two independent judges as a function of occupation categories. Among the 3,438 offers received, both independent judges agreed in their classification of 3,129 (91% of the total). From those agreed offers, 2,435 offers (71% of the total) were consistently classified in the target occupational categories (1 managers, 2 professionals, or 3 technicians) and therefore they were included in the analyses. In addition, 694 offers (20% of the total) were consistently classified in the (0) “others” occupational category as both judges estimated that they belonged to another occupational category or that it was not possible to classify them because the description was unclear. Finally, in 309 offers (9% of the total) judges did not reached an agreement. In these last two cases mentioned (offers classified as "other" and offers in which the judges did not reach an agreement), the offers were classified as missing values and therefore were not analyzed. We conducted a 2 (professional sector: female-dominated vs. male-dominated) x 2 (age: 24 vs. 38) x 2 (gender: female vs. male) ANOVA on each of the variables of interest. These variables were (a) the number of job offers received as a function of occupation category, (b) the annual gross salary, and (c) the number of long-term contracts. Table 3 shows the analyses of variance on these variables.

**Table 2:** Job offers received as a function of occupation category, annual gross salary, and temporariness (long-term contracts).

Professional sector	Age	Gender	Occupation categories				Missing values	Annual gross salary			Temporariness	
			Total*	Managers %	Professionals %	Technicians %		Total*	*M*	*SD*	Total*	Long-term contracts %
Female-dominated			1242	3.38	84.54	12.08	468	542	20,571	7,514	1,396	33.17
	24	Female	311	3.22	84.24	12.54	121	147	20,658	7,629	355	34.37
		Male	308	4.22	84.42	11.36	106	124	20,403	6,936	338	32.25
	38	Female	308	3.57	84.09	12.34	124	138	20,757	7,889	350	33.43
		Male	315	2.54	85.40	12.06	117	133	20,439	7,585	353	32.58
Male-dominated			1193	9.05	65.47	25.48	535	341	27,191	12,491	1,522	49.74
	24	Female	306	7.52	67.97	24.51	126	89	28,168	11,758	389	51.67
		Male	295	9.15	67.46	23.39	137	84	26,100	11,006	378	50.79
	38	Female	291	8.93	62.89	28.18	141	86	27,333	14,230	380	48.42
		Male	301	10.63	63.46	25.91	131	82	27,099	12,875	375	48.00
Total			2435	6.16	75.20	18.64	1003	883	23,128	10,255	2,918	41.44

Note.

*Total number of offers.

**Table 3:** ANOVA on the number of job offers received as a function of occupation categories, annual gross salary and number of long-term contracts.

Variables	Managers^a^			Professionals^a^			Technicians^a^			Annual gross salary^b^			Long-term contracts^c^		
	*F*	*p*	η^2^_p_	*F*	*P*	η^2^_p_	*F*	*p*	η^2^_p_	*F*	*p*	η^2^_p_	*F*	*p*	η^2^_p_
Professional sector	34.27	< .001	0.01	124.78	< .001	0.05	74.29	< .001	0.03	95.78	< .001	0.10	84.32	< .001	0.03
Age	0.16	0.685	0.00	1.46	0.227	0.00	1.15	0.283	0.00	0.01	0.912	0.00	0.85	0.357	0.00
Gender	0.73	0.394	0.00	0.05	0.823	0.00	0.60	0.438	0.00	1.13	0.287	0.00	0.35	0.554	0.00
Professional sector x Age	1.19	0.276	0.00	2.10	0.147	0.00	0.84	0.361	0.00	1.14	0.991	0.00	0.57	0.451	0.00
Professional sector x Gender	0.75	0.386	0.00	0.04	0.835	0.00	0.10	0.756	0.00	0.41	0.522	0.00	0.05	0.817	0.00
Age x Gender	0.26	0.610	0.00	0.10	0.745	0.00	0.00	0.969	0.00	0.43	0.512	0.00	0.06	0.811	0.00
Professional sector x Age x Gender	0.29	0.588	0.00	5.33	0.994	0.00	0.11	0.742	0.00	0.49	0.483	0.00	0.01	0.911	0.00

Note.

^a^ df = 1, 2427;

^b^ df = 1, 875;

^c^ df = 1, 2910.

As can be seen in Table 3, with respect to the number of job offers received as a function of occupation categories, the main effects of age and gender were not statistically significant. Neither were any of the interactions with age and gender, indicating that the manipulations of age and gender had no impact on the number of job offers received as a function of occupation category. However, we found a significant main effect of the professional sector. In the male-dominated sector, as compared to the female-dominated sector, candidates received significantly more offers for managers, *t (2427)* = 5.85, *p* < .001, *d* = 0.24; and technicians, *t (2427)* = 8.62, *p* < .001, *d* = 0.35; and less offers for professionals, *t (2427)* = -11.20, *p* < .001, *d* = -0.45.

In order to compare the salaries in the offers received, we analyzed the annual gross salary (see Table 3). Main effects of gender and age were not significant, and interactions were not observed. This indicates that the manipulations of gender and age had no direct impact on salaries. We found a significant main effect of the professional sector. The significant difference of professional sector, *t (875)* = 9.79, *p* < .001, *d* = 0.68, indicated that the annual gross salary in the male-dominated professional sector was higher than that in the female-dominated professional sector.

Finally, with respect to long-term contracts, the main effects of gender and age were not significant, nor were the interactions (see Table 3). This indicates that the applicants’ gender had no direct impact on the received offers. As in the previous analysis, we found a significant main effect of the professional sector. The significant difference observed as a function of the professional sector, *t (2910)* = 9.18, *p* < .001, *d* = 0.34, indicated that the number of long-term contracts in the male-dominated professional sector was higher than that in the female-dominated professional sector.

## Discussion

The present study explored whether job offers included in InfoJobs automated-job alerts varied as a function of gender. We used the correspondence testing procedure and examined different features of the offers included in the automated-job alerts as a function of whether gender was set as female or male. Several factors that potentially influence gender bias during personnel selection processes were controlled for, specifically, the professional sector (female-dominated vs. male-dominated), and the candidate’s age (24 vs. 38). Thus, we designed eight matched resumes in which we manipulated the gender, age, and professional sector of the candidates. In order to examine possible gender biases in the automated-job alerts, we explored the number of job offers received as a function of occupation category, salary, and number of long-term contracts. Although the correspondence testing procedure has been frequently used in the study of gender bias during the short-listing and hiring stages in personnel selection, we do not know of any previous study that has used this procedure to analyze the role of algorithms in the selection and forwarding of automated-job alerts to potential candidates. Because job platforms are increasingly used during job searching, and because those platforms involve the use of algorithms, research aimed at examining whether these initial stage algorithms exhibit biases are of great interest.

As the main result, we found no gender bias in the automated-job alerts received through InfoJobs, neither in a female-dominated professional sector (health) nor in a male-dominated professional sector (engineering) in Madrid during a period of approximately one month. In addition, we found no age bias. The automated-job alerts received did not differ significantly in relation to occupation category, salary, or long-term contracts as a function of gender or age. That is, manipulating the gender (female vs. male) as well as age (24 vs. 38) in the resumes did not give rise to significant differences in the number of job offers received as a function of occupation category, annual gross salary, or number of long-term contracts. Previous research found a gender bias during the final phases of personnel selection processes using InfoJobs [5] and other job platforms [26, 27]. Nevertheless, we have found no evidence of gender bias in the algorithm used by InfoJobs during the initial stage, that is, during the selection of which automated-job alerts are sent to which candidate. However, it should be noted that we did not observe any significant differences in the offers received as a function of age, which in our experiment had been correlated with experience, and therefore, different offers should have been expected, at least with respect to this variable. We suspect that the InfoJobs algorithm for this initial phase is possibly a very simple algorithm that does not send personalized offers as a function of gender, age, experience, or other personal attributes. This being so, it is not surprising that gender biases were not observed in this particular algorithm.

The fact that a gender bias was not found (not even differences due to experience) in this particular algorithm does not imply that potential biases are negligible. We want to highlight the need to design controlled experiments to investigate the behavior of algorithms and possible biases in automated-job alerts. This may be of great interest given the extensive use of algorithms and the already reported gender biases in the selection processes in the algorithms used by Google Ads [41] and Amazon [42]. Indeed, running these experiments is how we may have to discover whether an algorithm is or is not biased, and the criteria that an algorithm is using when classifying candidates.

Additionally, our results show that the professional sector is a factor that generates significant differences in the variables studied. Candidates in the male-dominated professional sector received a greater number of offers for managers and technicians and fewer offers for professionals as compared to those in the female-dominated professional sector. It should be clarified that the significant effect of the professional sector in the present research was not due to a gender bias in the algorithm but to the offers published by the companies. These findings are partly congruent with previous research showing that masculinization occurs to a greater extent in highly qualified positions [2, 8, 21, 26]. Data from OECD countries, including Spain, show that high-growth sectors such as science, technology, and engineering are still dominated by men and show salary differences between men and women [3]. In addition, in the male-dominated professional sector in our study, the annual gross salary of the offers received was higher than in the female-dominated professional sector. These findings are consistent with the results of international reports highlighting that one of the causes of the pay gap between men and women is due to the imbalance of their participation in different types of industries. For example, the World Bank [3, 4] documents substantial salary differences between male-dominated and female-dominated occupations. In sum, women earn less than men even when controlling for occupation, and the gender distribution through different types of work reinforces income disparities and the vulnerability of women. As for the long-term contracts, a greater number of offers with long-term contracts were received in the male-dominated professional sector as compared to the female-dominated one. In line with these results, it has been documented that, on average, temporary employment rates for women are higher than for men in the OECD countries. Women are more likely to work in temporary and part-time jobs than men [3, 55]. In sum, the offers received from InfoJobs reflected higher salaries and more offers of long-term contracts in male-dominated jobs than in female-dominated jobs, a phenomenon widely documented in previous literature (e.g. [10, 26]). These differences have been called the *precariousness of feminized work* and are consistent with the data of the labor market [9]. In addition, the pay gap as a function of the professional sector is one of the causes of the *gender pay gap* and the *average gender overall earnings gap* [10, 56–58].

This research presents some limitations that should be mentioned: By restricting the professional sector and geographical area, we significantly reduced the number of job offers received since we only had access to some of the total number of offers available in InfoJobs. Furthermore, in this research, we only examined the health sector as the female-dominated sector and the engineering sector as the male-dominated sector, but it would be interesting to replicate these results in other gendered professional sectors as well. Another limitation is that we reduced the number of job offers received and analyzed in terms of occupation category. The reason is that we wanted to examine only those offers that conformed to the CNO-11 [51], a reliable and standard criterion that coincides with those of the International Labour Organization [52] and by the European Commission [53]. Furthermore, by selecting only the professional sectors of health and engineering, we were only able to select offers that corresponded to the first three categories, since it was in these first three categories where offers from both sectors concurred. It could be valuable for future research to analyze other professional sectors, other geographical areas, and other occupation categories. In any case, our data are publicly available, so anyone interested could also further exploit the information included within the job alerts that we received.

The results of this study have several implications. Although we did not find a gender bias in the InfoJobs automated-job alerts, when we started the study in 2019 gender was still a required field during the registration process. Today, however, it is no longer a mandatory field. Another job platform commonly used in Spain is Infoempleo.com. Similarly, in Infoempleo gender information was requested during the registration process until recently. The fact that gender was requested suggests that it was being used. In this vein, we also reviewed other job platforms in Spain such as upwork.com, indeed.es, and linkedin.es and found that gender information is not requested in 2021 during the registration process.

Because there is prior evidence that information about the gender of candidates affects both algorithms and employers, many have recommended a blind selection process [59, 60] in which the gender of the candidates is unknown to the person and the machine in charge of the selection process. Anonymous procedures can be easily conducted through job platforms, especially in the early stages of the selection process, by eliminating the gender information. In this regard, many job platforms, such as InfoJobs and Infoempleo, have recently changed their policies concerning the mandatory nature of gender information. In fact, previous studies show that women get higher callback rates when anonymous job applications are used (see [18] for a review).

In addition, it has also been shown in the literature that it is necessary to use non-sexist language in order to not reinforce discrimination. Algorithms learn patterns from the data that they receive, including language-related data. For example, we found that the language describing the offers exhibits a gender bias (e.g. the search on the feminized sector for the term "enfermera” [nurse] in femenine produced 30% more offers than in masculine “enfermero”; while in the masculinized sector "ingeniero” [engineer] in masculine produced 18% more offers than in feminine “ingeniera”). To the extent that the use of language exhibits bias in the job platforms’ data, there is a risk of amplifying them through the use of algorithms (e.g. [39]).

Also, due to the concerns about machine learning systems raised from some sounding studies, a number of researchers have developed technical proposals and recommendations on how to reduce possible gender biases [61–64]: Firstly, AI-powered systems should be applied with transparency. The code of the algorithm and the implementation process should be open to allow auditing, regulation, and suggestion of improvements [65]. In addition, transparency for users has also been suggested by offering the profile information used to select the content that will be shown [41]. Among the technical proposals, there are approaches to introduce learning models for the control and protection of discrimination [63], algorithms for calibration and correction bias [66], and tools to audit data using significance tests [41]. The second recommendation is related to the team involved in AI research, development, and implementation. There should be a racial, social, and gender diversity of the team and more effective collaboration between experts from different disciplines (e.g., engineers, lawyers, psychologists), as well as collaboration among experts and segments of the affected population [63]. In fact, in most countries, women are under-represented in science and engineering positions [9]. Finally, there is a need to increase training and awareness of employers and programmers about the impact of bias in algorithms to strive to change their practices, aligning their economic and performance incentives with non-discrimination objectives [61]. Many employers are changing their recruitment practices by including AI in order to reduce bias. However, they are unaware that they might be introducing new forms of biases. Moreover, when discrimination results from the unintended and unknown use of the property of an algorithm rather than from conscious choice, it may be difficult to identify the source of the problem.

## Conclusions

Regardless of the particular results that we observed in the particular algorithm that we studied, our research shows that procedures that are commonly used in the social sciences to study human behavior and cognition (i.e., controlled experiments; correspondence testing procedure) can be successfully applied to the audit of algorithms. In particular, we have adapted the standard procedure of correspondence testing to auditing an algorithm used for selecting and sending automated-job alerts in a leading job platform. More generally, we can also conclude that designing controlled experiments inspired in the methods of Experimental Psychology is proving to be a successful strategy to audit the behavior, criteria, and biases of proprietary and opaque algorithms. Consequently, companies have to know that opaque algorithms that affect people’s lives can become subject to behavioral research.
